# *Artemisia abrotanum* and *Symphytum officinale* Polyphenolic Compounds-Rich Extracts with Potential Application in Diabetes Management

**DOI:** 10.3390/metabo13030354

**Published:** 2023-02-27

**Authors:** Elena Neagu, Gabriela Paun, Camelia Albu, Sandra Ana-Maria Victoria Eremia, Gabriel Lucian Radu

**Affiliations:** National Institute of Research and Development for Biological Sciences, 296 Splaiul Independentei, 060031 Bucharest, Romania

**Keywords:** *Artemisia abrotanum*, *Symphytum officinale* polyphenolic compounds-rich extracts, bioactive compounds, antidiabetic potential

## Abstract

Lately, there has been increased interest in the development of phytochemical alternatives for the prevention and treatment of type 2 diabetes, the alternatives that are able to reduce or prevent glucose absorption by inhibiting digestive enzymes. In this context, this study aims to analyze the inhibitory α-amylase and α-glucosidase activities of *Artemisia abrotanum* and *Symphytum officinale* polyphenolic compound-rich extracts obtained by membrane technologies (micro- and ultrafiltration). Polyphenols and flavones content, HPLC-MS polyphenolic compounds profiling, antioxidant activity, and cytotoxic potential of these herbs were determined. Major phenolic acid compounds were chlorogenic acid, ellagic acid, caffeic acid, and rosmarinic acid. The flavone content was higher in the case of *A. abrotanum* extracts, and the major compounds were rutin and umbelliferone. The polyphenolic-rich extract of *A. abrotanum* had the highest quantities of polyphenols, 977.75 µg/mL, and flavones, 552.85 µg/mL, as well as a pronounced α-amylase inhibitory activity (IC50 1881.21 ± 1.8 mg/mL), a value close to acarbose inhibitory activity (IC50 1110.25 ± 8.82 mg/mL) that was used as the control for both enzymes. The α-glucosidase inhibitory activity was higher for both herb extracts, more pronounced for *S. officinale* polyphenolic-rich extract (IC50 291.56 ± 2.1 mg/mL), a value higher than that of acarbose (IC50 372.35 ± 3.2 mg/mL). These plants show potential as a complementary therapy for type 2 diabetes management.

## 1. Introduction

Usage of nutraceuticals in the treatment of chronic diseases is encouraged in developed countries due to the side effects of synthetic drugs. The treatment based on natural remedies seems to offer more opportunities in the treatment of such conditions. Medicinal herbs were used widely because they contain biologically active compounds with a synergistic effect and have reduced side effects and lower costs compared to allopathic drugs [1,2].

Diabetes mellitus is a serious chronic endocrine condition with multiple etiologies and increasing incidence due to obesity and aging. Over time, this condition leads to severe complications, such as retinopathy, neuropathy, nephropathy, atherosclerosis, and vascular complications that increase cardiovascular risk up to heart attack [3,4,5]. Type 1 diabetes, which is insulin-dependent, is an autoimmune disease caused by the destruction of pancreatic β-cells, which secrete little or no insulin. In non-insulin-dependent type 2 diabetes, the pancreas secretes insulin, but it is not used efficiently in the cells. About 90–95% of diabetes cases are type 2 [6].

There are many oral drugs used to treat type 2 diabetes, for example, metformin [7] is the most commonly used; then, there are drugs in the sulfonylurea, thiazolidinedione, and α-glucosidase inhibitor classes [8]. However, they present a series of adverse side effects, such as liver and cholecystic lesions, diarrhea, nausea, vomiting, lactic acidosis [9], hypoglycemia, weight gain, skin reactions, porphyria [10], and gastrointestinal disorders [11].

A therapeutic alternative for diabetes treatment consists of the reduction of post-prandial hyperglycemia, which can be realized by delaying glucose absorption via α-glucosidase and α-amylase inhibiting [12]. The α-amylase hydrolyzes starch in maltose and glucose, while α-glucosidase hydrolyses maltose and sucrose with the release of glucose. Inhibiting these enzymes, carbohydrate digestion and glucose absorption are delayed and therefore, postprandial hyperglycemia is reduced [3,13]. Many attempts were made to find new and effective natural alternative inhibitors to these enzymes and to produce food additives or compounds used against diabetes [14]. Many studies have highlighted those bioactive compounds, especially polyphenols, which have the ability to inhibit these enzymes. For this reason, in this work, we studied the α-amylase and α-glucosidase inhibitory capacity of polyphenolic compound-rich extracts from *Artemisia abrotanum* and *Symphytum officinale*. These plants were chosen for this study because they are widespread in Europe and not at all studied from the point of view of inhibiting these enzymes.

*Artemisia abrotanum* (southernwood) (family Asteraceae) is a medicinal herb used as an antiseptic, anti-inflammatory astringent, antipyretic, tonic, and for the treatment of respiratory diseases. It has also been used against cancer, cough, fever, and tumors [15]. It also shows antibacterial, antifungal, and antiparasitic effects [16].

*Symphytum officinale* (comfrey) (family Boraginaceae) is a herb used in folk medicine. *S. officinale* leaves and the roots were used in the treatment of gastrointestinal tract ulcerations, bones, and respiratory diseases [17,18,19]. *S. officinale* contains allantoin, carotene, hydroxycinnamic acid, and essential oils. Comfrey also contains hepatotoxic pyrrolizidine alkaloids, so its long-term consumption is not recommended [20,21].

Special attention is paid to the extraction and concentration of polyphenolic compounds since they are thermolabile and prone to chemical changes. The membrane technologies—microfiltration, ultrafiltration, and nanofiltration—are some of the most modern and effective methods of separation and concentration of bioactive compounds from plant extracts. Processing is performed at mild temperatures, maintaining the original characteristics of the products processed, with minimal loss of vitamins, polyphenols, proteins, and other thermolabile compounds [22,23,24]. Recent studies demonstrated the efficiency of ultrafiltration and nanofiltration for the concentration of bioactive compounds, such as anthocyanins, flavonoids, oligosaccharides, and phenolic compounds from vegetable extracts [25,26,27].

## 2. Materials and Methods

### 2.1. Materials

All reagents have been purchased from Fluka (Buchs, Switzerland), Roth (Carl Roth GmbH, Karlsruhe, Germany), and Sigma (Sigma–Aldrich, Schnelldorf, Germany).

### 2.2. Plant Material

*Artemisia abrotanum* was harvested from Rodna, Bistrita-Nasaud district was identified by Sorana Hentea and deposited at Cluj Herbarium (voucher specimen nr. 210.240). The leaves of *Symphytum officinale* were collected from Valea Morii, Feleac, Cluj district, identified by Sorana Hentea, and deposited at Cluj Herbarium (voucher specimen nr. 657369).

### 2.3. Obtaining and Concentrating Extracts

The ethanol extracts were obtained as follows: 10 g of the dry plant (aerial parts) was added to 100 mL of 50% (*v*/*v*) EtOH, were mixed, then were sonicated for 1 h at 25 °C, and finally, filtered through a Whatman filter (125 mm, Blue–Slow).

First, the extracts were micro-filtrated through a Millipore membrane (0.45 µm pores) for purification. Then, the extracts were concentrated by ultrafiltration through Millipore membranes with a cut-off of 1000 Da. The concentration ratio (ratio of permeate volume to concentrate) was 3:1. For microfiltration and ultrafiltration, a KMS Laboratory Cell CF-1 installation was used.

### 2.4. Phytochemical Characterization

#### 2.4.1. Polyphenolic Compound Content Determination

Polyphenolic compound content determination was done using Folin–Ciocalteu method [28]. Thus, 2.5 mL extract and 2.5 mL Folin–Ciocalteu reagent were mixed, shaken, and filtered; then, 1 mL of the filtrate was added to 9.5 mL sodium carbonate 20% and absorbance reading at 660 nm after 2 min. The polyphenolic compound content in the extracts was determined based on a gallic acid calibration curve.

#### 2.4.2. Flavonoid Content Determination

Flavonoid content determination was achieved using the spectrophotometric method described by [29]. Thus, 5 mL of sample and 7.5 mL of MeOH were mixed and then filtered. Afterward, 1 mL filtrate was mixed with 1 mL sodium acetate solution (10%); 0.6 mL of aluminum chloride hexahydrate solution (2.5%) and 0.5 mL MeOH were added. The absorbance reading was performed at 430 nm after 15 min. The flavonoid content was determined by a rutin calibration curve. 

#### 2.4.3. HPLC Analysis

The chromatographic analysis has been realized with an HPLC SHIMADZU system (C18 Nucleosil 3.5, 4.6 × 50 mm Zorbax column). An MS detector, LCMS-2010 detector (liquid chromatograph mass spectrometer), with an ESI interface and negative ionization mode, was coupled to the system. All reagents used have been of analytical purity. Ethanolic stock solutions, 1 mg/mL of ellagic acid, rutin, caffeic acid, quercetin, gallic acid, rosmarinic acid, chlorogenic acid, apigenin, quercetin 3-β-D-glucoside, luteolin, kaempferol, and umbelliferone were used as standards. The mobile phase consisted of water (solvent A) and acetonitrile (solvent B), adjusted to pH 3.0 with formic acid. Separation of phenolic compounds was achieved using binary gradient elution as follows: between 0.01–20 min; the initial 5% solvent B was increased to 30% and from 20.01–40 min maintained at 30%; then, at 50% between 40.01–50 min, returning to 5% between 50.01–52 min and held at 5% for 52.01–70 min. The flow rate was 0.1 mL/min between 0–5, 15.01–35, and 60.01–70 min, and 0.2 mL/min from 5.01–15 and 35.01–60 min. We used Alecu’s HPLC method to analyze polyphenols [30]. The mobile phase consisted of water (solvent A) and acetonitrile (solvent B), adjusted to pH 3.0 with formic acid. Separation of phenolic compounds was achieved using binary gradient elution as follows: between 0.01–20 min, the initial 5% solvent B was increased to 30%, from 20.01–40 min maintained at 30%, then at 50% between 40.01–50 min, returning to 5% between 50.01–52 min and held at 5%, 52.01–70 min. The flow rate was 0.1 mL/min between 0–5, 15.01–35, and 60.01–70 min, and 0.2 mL/min from 5.01–15 and 35.01–60 min. For qualitative analysis, we used acquisition mode, SCAN of MS, [M-H]^−^: 50-700, and UV scanning spectra. Ethanolic stock solutions, 1mg/mL of umbelliferone (Sigma, H24003), gallic acid (Fluka, 48630), caffeic acid (Sigma, C0625), apigenin (Fluka, 10798), luteolin (Sigma, 72511), kaempferol (Sigma, 60010), ellagic acid (Fluka, 45140), quercetin (Sigma, Q4951), chlorogenic acid (Sigma, C3878), rosmarinic acid (Sigma, 536954), quercetin 3-β-D-glucoside (Sigma, 17793), and rutin (Sigma, 78095) were used as external standards for quantitative analysis. Using the SIM (selected ion monitoring) mode ([M-H]-: 161, 169, 179, 269, 285, 301, 353, 359, 463, and 609), the corresponding peaks of the polyphenols compound fragment ions were obtained, and were in perfect accordance to the data resulted from PDA chromatogram. 

### 2.5. Antioxidant Assays

The antioxidant activity has been analyzed through two methods.

#### 2.5.1. DPPH Radical Scavenging Activity

Briefly, 100 μL extract having different concentrations (3 mg/mL, 1.5 mg/mL, 0.75 mg/mL, 0.3 mg/mL) was mixed with 1 mL 2,2-diphenyl-1-picrylhydrazyl (DPPH) solution (0.25 mM) and 1.9 mL methanol [31]. The extracts’ scavenging activity was calculated through the reading of absorbance at 517 nm after 3 min and was determined by the formula:

radical scavenging activity (%) = [(AB − AA)/AB] × 100,

where AB = control absorbance, and AA = sample absorbance.

The IC_50_ (extract concentration which produces 50% radical scavenging activity) values were determined by linear regression analysis. Significant statistical differences were considered *p* < 0.05.

#### 2.5.2. Reducing Power Activity (Iron (III) to Iron (II) Reduction)

Reducing power has been analyzed using Berker’s method [32]. Thus, a 0.1 mL sample with 2.5 mL of sodium phosphate buffer (200 mM/L, pH 6.6) and 2.5 mL potassium ferricyanide (1%) was mixed. The mixture was vigorously agitated, then heated for 20 min at 50 °C; then, 2.5 mL trichloroacetic acid (10% *w*/*v*) was added after incubation. Finally, into the mixture, 2.5 mL deionized water and 0.5 mL ferric chloride (0.1%) were added. The absorbance has been read at 700 nm. The EC50 (the concentration corresponding to an absorbance of 0.5) values were calculated through linear regression analysis. Significant statistical differences were considered *p* < 0.05.

### 2.6. Enzymes Inhibition Activity

The *Artemisia abrotanum* and *Symphytum officinale* extracts were analyzed for the α-amylase and α-glucosidase inhibition activity.

#### 2.6.1. α-Amylase Inhibition

The α-amylase inhibition assay was performed with modified Ranilla’s method [33]. Thus, 100 μL of the sample was mixed with 250 μL α-amylase from hog pancreas (EC 3.2.1.1) (0.5 mg/mL) in sodium phosphate buffer (0.02 M, pH 6.9) and was incubated at 37 °C for 20 min; then, 250 μL starch solution (1%) was added; then, the mixture has been reincubated at 37 °C, 30 min. After that, 500 μL dinitrosalicylic acid (DNS) was added; then, the reaction mixture was kept in the boiling bath for another 5 min. Then, 5 mL of distilled water was added to the mixture. Finally, the absorbance reading was performed at 540 nm using a UV-visible spectrophotometer (Jasco-V630). As a positive control, acarbose was used. The calculations were done by the formula:

$$\%\;\mathrm{Amylase}\;\mathrm{inhibition}=\frac{\Delta A_{control}-\Delta A_{sample}}{\Delta A_{control}}\;\times \;100$$

IC_50_ (concentration of extract, which produces 50% enzyme inhibition) values have been determined through the linear regression analysis. Significant statistical differences were considered as *p* < 0.05.

#### 2.6.2. α-Glucosidase Inhibition

The α-glucosidase inhibitory activity was realized by Queiroz et al. method with minor modification [34]. Briefly, 120 μL α-glucosidase (EC 3.2.1.20) (0.5 U/mL) was mixed with 720 μL sodium phosphate buffer (0.1 M, pH 6.9) with 60 µL extract and heated at 37 °C for 15 min. Then, a 120 μL p-nitrophenyl-α-D-glucopyranoside (5 mM/L) solution was added. The mixture was heated at 37 °C for 15 min. Finally, the absorbance reading was performed at 405 nm. The positive control was acarbose. The calculations were done by the formula:

$$\%\;\mathrm{Glucosidase}\;\mathrm{inhibition}=\frac{\Delta A_{control}-\Delta A_{sample}}{\Delta A_{control}}\;\times \;100$$

IC50 values were determined by the linear regression analysis. Significant statistical differences were considered as *p* < 0.05.

### 2.7. Testing Extracts’ Cytotoxic Activity In Vitro

The cytotoxic activity of *A. abrotanum* and *S. officinale* extracts was analyzed on NCTC clone 929—a mouse fibroblast cell line. These were procured from the European Collection of cell Culture (Sigma–Aldrich, St. Louis, MI, USA). All materials used for the culture experiments were obtained from Sigma–Aldrich (SUA): PSN (100 mg/mL penicillin, 100 mg/L streptomycin, and 500 mg/L neomycin), MEM (Minimum Essential Medium), Giemsa, and Neutral Red from Merck (Germany).

- *Cell Viability*

NCTC has been grown in MEM with PSN and 10% fetal bovine serum (FBS). The NCTC line was inoculated at 4 × 104 cells/mL density, then incubated in the wet atmosphere (5% CO2, 24 h) at 37 °C to allow the adherence of the cells. The untreated cells (Mc) were used as the control, and cells cultured in 100 µM H2O2 were used as the positive control. Then, the culture medium was changed by the same medium, with several ex-tract concentrations (50, 100, 500, and 1000 µg/mL). The cells were cultured in standard conditions (24, 48, and 72 h).

- *Neutral Red Test*

The cell viability was analyzed by Borenfreund’s Neutral Red (NR) method [35]. The cellular line of fibroblast cultures (NCTC) was used to test cytotoxicity. After removal from the culture medium, the cells were washed with phosphate buffer. Then, 0.5 mL of freshly prepared NR solution (10 µg/mL) was added to each well and incubated at 37 °C for 3 h. Subsequently, the NR solution was removed and fixed with a 1% formol solution for 3 min. The plates were stirred at room temperature for 15 min and then transferred to a microplate reader (Tecan Microplate Reader). Spectrophotometric determinations were performed at 540 nm. All experiments were done in triplicate.

The viability of cells has been determined by the equation:

% cell viability = (As/Ac) × 100,

where Ac = cellular line control, and As = the cellular line with various extracts. Significant statistical differences were considered *p* < 0.05.

- *Light Microscopy*

The NCTC culture cell was fixed in methanol and then Giemsa dye-marked. Cells were observed 72 h after the addition of extracts. The images were taken with a Zeiss AxioStar Plus microscope. Untreated cells (Mc) were used as a control, and cells cultured in the presence of curcumin (1 mM) were used as a control.

### 2.8. Statistical Analysis

The tests have been done in triplicate. For the statistical analysis, the software Microsoft Office Excel 2007 was used, and the standard deviation (STDV) was less than 10%. Statistical analysis was performed using Student’s *t*-test, and the values were considered significant when *p* < 0.05. The test was used to evaluate the relationship between antioxidant activity and biological compound content (polyphenols, flavonoids) as well as the relationship between enzyme inhibition activity and biological compound content.

## 3. Results and Discussions

### 3.1. Polyphenols and Flavonoid Content Determination

The content of total polyphenols was in the tested samples between 667.58–977.75 μg/mL and was similar for the two plants, slightly higher in the case of *Artemisia abrotanum* extracts. The highest content of polyphenols, 977.75 μg/mL, was determined in the concentrated extract of *A. abrotanum*. Regarding the flavonoid content, the *A. abrotanum* extracts showed significantly higher amounts than the *S. officinale* extracts. The highest flavonoid content—552.85 μg/mL was determined in the same concentrated extract of *A. abrotanum*. The results obtained are shown in Table 1.

*A. abrotanum* extracts showed higher amounts of polyphenols and especially flavonoids than *S. officinale* extracts; the concentrates were enriched in polyphenols and flavonoids compared to the initial extracts (microfiltration); *A. abrotanum* concentrate showed the highest concentration of polyphenols and flavonoids.

More biological activities are attributed to phenolic compounds, such as antioxidant activity, antimicrobial anti-inflammatory, anticancer, and coronary protective activities [36,37]. Recently, it was found that phenolics can contribute to type 2 diabetes treatment because they play a role in amylase inhibition [38]. Numerous studies showed that antioxidant activity is related to compounds, such as polyphenols, flavonoids, isoflavonoids, flavonoids, catechins, and vitamins (ascorbic acid, α-tocopherol, β-carotene) [39].

### 3.2. HPLC Analysis

**Figure 1:**
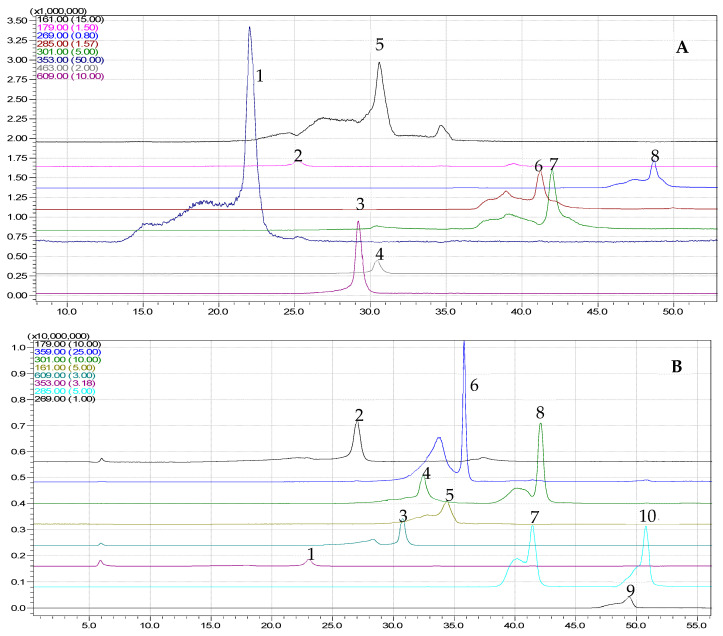
Chromatograms obtained for *A. abrotanum* polyphenolic compound-rich extract (**A**) and *S. officinale* polyphenolic compound-rich extract (**B**) by HPLC-MS ((**A**), 1-chlorogenic acid, 2-caffeic acid, 3-rutin, 4-quercetin 3-β-D-glucoside, 5-umbelliferone, 6-luteolin, 7-quercetin, 8-apigenin peaks, respectively, (**B**), 1-chlorogenic acid, 2-caffeic acid, 3-rutin, 4-ellagic acid, 5-umbelliferone, 6-rosmarinic acid, 7-luteolin, 8-quercetin, 9-apigenin, and 10-kaempferol peaks).

The HPLC-PDA-MS method was applied to the identification of polyphenolic compounds analytes by retention time method, SCAN mode of MS, and UV spectra, and the results obtained regarding the concentrations of the compound from the tested plant extracts are given in Table 2 and Figure 1. Good linearity of the method with the studied concentration range (0.1–50 µg/mL) was observed.

The results obtained regarding the tested plant extracts are given in Table 2.

From Table 2, it can be observed that the polyphenolic compounds are concentrated by ultrafiltration. A total of 12 compounds were identified and quantified in both extracts. The *A. abrotanum* polyphenolic compound-rich extract had higher quantities of rutin (10.57 µg/mL), umbelliferone (17.03 µg/mL), and chlorogenic acid (103.47 µg/mL), while *S. officinale* polyphenolic compound-rich extract had higher quantities of caffeic acid (9.42 µg/mL), ellagic acid (43.79 µg/mL), and rosmarinic acid (136.140 µg/mL). As shown in other studies, chlorogenic acid and rutin have also been identified as major constituents in Artemisia ethanolic extract from other species [40,41]. Although there are few studies on the polyphenolic compounds profile of *S. officinale* extracts, rosmarinic and caffeic acid were also found to be major polyphenols by Sowa et al. [42], Trifan et al. [43], and the presence of caffeic acid was also reported by Nastić et al. [44].

### 3.3. Antioxidant Activity Determination

For the assessment of the antioxidant potential of extracts, a single assay method is not sufficient. Therefore, for antioxidant activity evaluation of the studied herbal extracts, we used two different methods, one used organic radical producers DPPH, and the other used metal ion for oxidation (Fe).

The antioxidant activity of microfiltrate and polyphenolic compound-rich extract from both plants was evaluated using two assays, reducing power and DPPH. The results obtained are shown in Table 3.

It can be observed from Table 3 that the *A. abrotanum* extract had higher antioxidant activity than *S. officinale* by both analysis methods. The *A. abrotanum* extract had a more reducing power activity, EC_50_ 92.14 mg/mL, than the ascorbic acid, EC_50_ 1110.15 mg/mL (used as control), while *S. officinale* extract had similar reducing power with the control. 

Considering the DPPH radical scavenging activity, both plant extracts showed smaller activity values than ascorbic acid does, except the *A. abrotanum* concentrate (IC_50_—9.31 mg/mL) had higher activity than the control activity—IC_50_—11.76 μg/mL. Moreover, the highest content of polyphenols and flavonoids was obtained for *A. abrotanum* concentrate.

DPPH analysis is one of the best-known, accurate, and frequently employed methods for evaluating antioxidant activity. The effect of antioxidants on DPPH is thought to be due to their hydrogen-donating ability.

Reducing power is also widely used in evaluating the antioxidant activity of plant polyphenols. The reducing power is generally associated with the presence of reductants, which exert antioxidant action by breaking the free radical chains by donating a hydrogen atom. 

An antioxidant is a compound that exhibits the capacity to transfer electron and/or hydrogen atoms during free radical neutralization [45,46]. The antioxidant ability of phenolic compounds depends on the number of hydroxyl groups in the ring structure and their arrangements.

A good correlation (*p* < 0.05) was observed between the content of polyphenols and flavonoids and the radical scavenging activity and the reducing power activity of the analyzed extracts for both studied herbs. Other studies have also indicated significant correlations between the polyphenolic compound content and antioxidant capacity, indicating that polyphenols were the main constituents in the tested medicinal plants [47]. The antioxidant properties of polyphenols and flavonoids derive from their redox properties, metal ion chelating activity, and singlet oxygen scavenging ability [48]. Therefore, the composition of the extracts could lead to a synergistic and even stronger antioxidant capacity.

Polyphenols, through their antioxidant effect, can have an important role in diseases generated by oxidative stress. This is due to the disturbance of the equilibrium between the appearance of free radicals and the body’s ability to eliminate them. The appearance of many ailments, such as diabetes mellitus, cancer, Alzheimer’s, etc., relates to these radicals that can be scavenged by antioxidants [31,49].

### 3.4. Testing the Cytotoxic Activity of the Extracts

**Figure 2:**
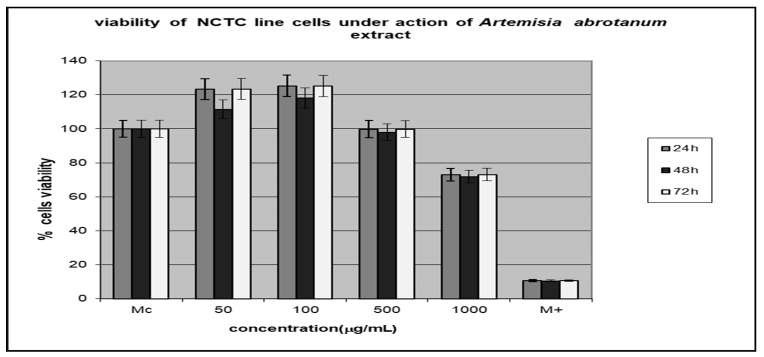
The viability of NCTC cell lines under the action of *A. abrotanum* extracts.

**Figure 3:**
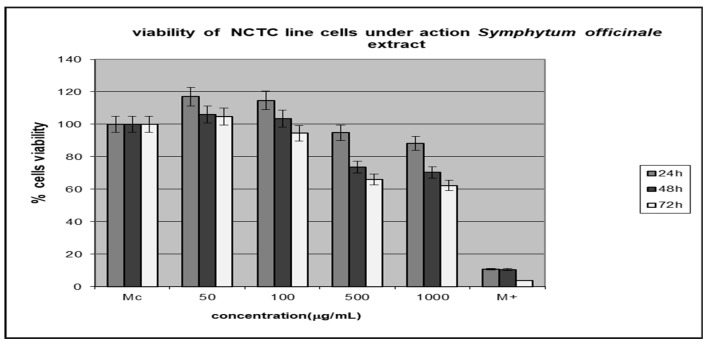
The viability of NCTC cell lines under action *S. officinale* extracts.

In order to increase the reliability of the obtained results and to avoid underestimating the toxicity of plant extracts, the cytotoxic effect of the *A. abrotanum* and *S. officinale* extracts were examined using NCTC cells. The extract cytotoxicity has been analyzed by testing the cellular viability in percent, and the results are presented in Figure 2 and Figure 3. The data represent the means ± SD of triplicate samples of three independent experiments.

The *A. abrotanum* polyphenolic compound-rich extract was tested in the concentration range of 50–100 µg/mL, and it showed a non-cytotoxic effect at 500 µg/mL and a little cytotoxic effect at 1000µg/mL.

The *S. officinale* polyphenolic compound-rich extract had a non-cytotoxic effect in the concentration range of 50–100 µg/mL and was slightly cytotoxic starting with 500 µg/mL.

The extracts’ cytotoxicity has been dependent both on the extract concentration and the time interval, and it has been higher than 40% in the case of *S. officinale* tested at the highest concentration and longest time of action. 

#### Morphological Characterization of Cell Lines

The cytotoxic effect of the studied polyphenolic compound-rich extracts in two different doses, 100 µg/mL and 1000 µg/mL, for 72h was shown in the photomicrographs.

**Figure 4:**
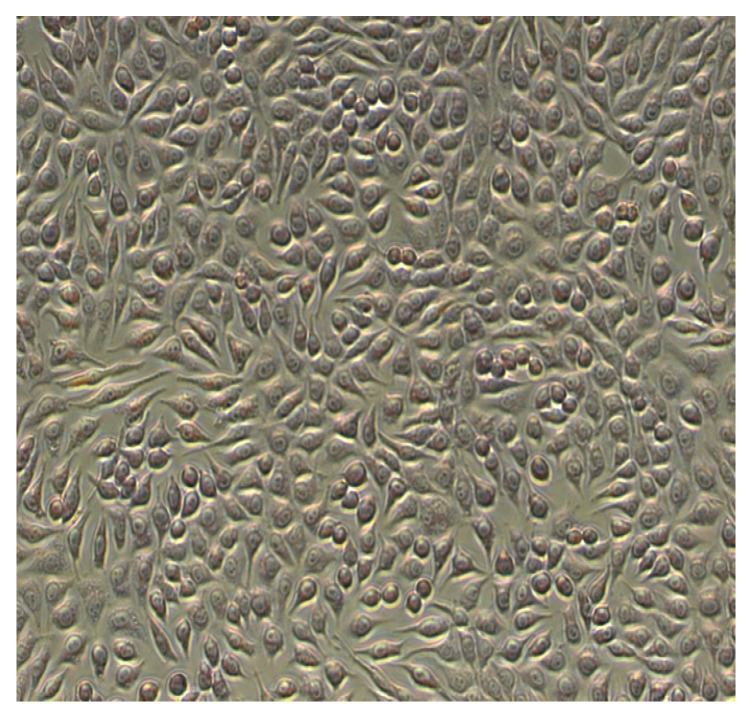
NCTC control, 72 h.

The NCTC cell line presents fibroblast-type morphology—the cells have a spindle shape with a large spherical nucleus, fine cytoplasmic granulation, and two–three nucleoli. At 72 h, the cell line was confluent. (Figure 4).

**Figure 5:**
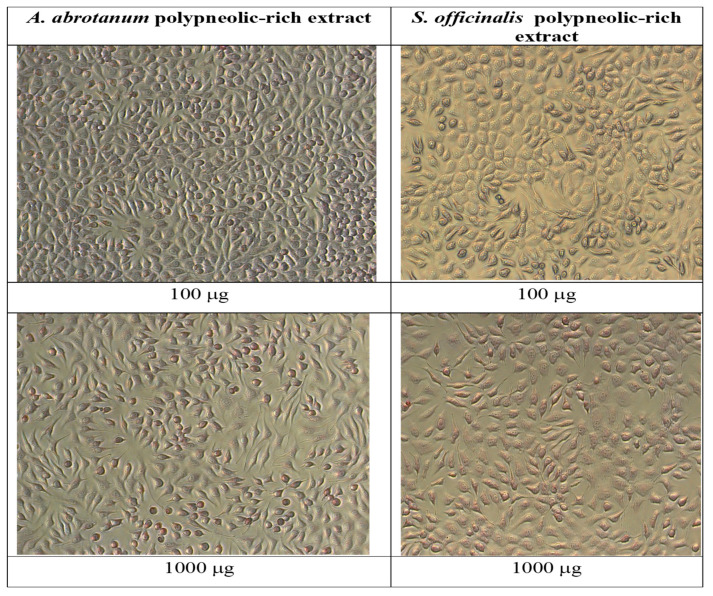
Normal NCTC cell line.

The NCTC cells exposed to *A. abrotanum* polyphenolic compound-rich extract were similar to the cellular line, did not present any morphological modifications, and the cellular density was comparable to the control. The culture was dense, the cells covering the whole well surface in the concentration range of 50–500 µg/mL, with a weak decrease at concentrations higher than 1000 µg/mL (Figure 5).

The structure of the NCTC cells exposed to *S. officinale* polyphenolic compound-rich extract had morphological modifications compared to the control, which became obvious at 1000 µg/mL (Figure 4).

### 3.5. α-Amylase and α-Glucosidase Inhibition Activity

Recently, more and more natural sources have been investigated to find remedies to lower glucose production and absorption in the intestine [50]. An effective method of controlling diabetes is to inhibit α-amylase and α-glucosidase activities. These enzymes are involved in the starch cleavage in sucrose and maltose, with glucose release, hence decreasing so glucose absorption [51]. In this study, the potential enzyme inhibitors of two herbs from Romania—*A. abrotanum* and *S. officinale*—were investigated; these herbs have not been researched before from this point of view. Data obtained regarding the α-amylase and α-glucosidase inhibition activity by the analyzed extracts are shown in Table 4. 

The *A. abrotanum* extracts have stronger α-amylase inhibitory activity than the *S. officinale* extracts, particularly the concentrated extract—IC_50_ = 1881.21 ± 1.8 mg/mL and close to the value of acarbose used as control. Regarding the α-glucosidase inhibitory activity, both extracts have high activity, close to acarbose activity (IC_50_ = 372.35 ± 3.2 mg/mL) used as control, namely IC_50_ = 291.56 ± 2.1 mg/mL for *S. officinale* extract and IC_50_ = 1171.16 ± 6.5 mg/mL for *A. abrotanum* extract, respectively. The concentrated extracts had higher activities than the extracts obtained by microfiltration. Moreover, a good correlation was noticed between the polyphenols and flavonoid content and the α-amylase and α-glucosidase inhibitory activity of both tested herbs. In conclusion, the tested extracts had a strong α-glucosidase inhibitory activity and a weaker α-amylase inhibitory activity in agreement with the literature data [51,52].

The previous studies had assigned the high α-amylase and α-glucosidase inhibitory effect of the extracts to the high content of polyphenols and flavonoids [53].

Chlorogenic acid, present in high quantities in *A. abrotanum* and in many other plants, has antioxidant, antidiabetic, and other pharmacological activities. Some previous studies showed hypoglycemic and hypolipidemic effects of chlorogenic acid and rutin, specifically in their possible application in the prevention and treatment of diabetes mellitus [53,54,55].

Rosmarinic acid, the main compound in S. officinalis, is a potent antioxidant compound. Rosmarinic acid-rich extract from herbal plants has shown significant anti-diabetic effects in diabetes-induced animal models in vivo and insulin-like effects in insulin target cells in vitro. Recent studies indicated that rosmarinic acid acts through various mechanisms, such as inhibition of α-amylase and α-glucosidase, suppression of insulin-resistant HepG2 cells through activation of AMPK phosphorylation, as well as acting as an antioxidant [56].

The flavonoids reduce the oxidative stress at the level of beta cells from pancreatic, thus reducing the risk of type 2 diabetes apparition [57,58]. Flavonoids, such as rutin, found in *A. abrotanum* extracts, quercetin, and kaempferol, found in both types of extracts, apigenin, and myricetin, have potential protective properties against cardiovascular disease and diabetes, and quercetin is a good α-amylase inhibitor [59]. Umbelliferone, found in *A. abrotanum* extracts, belongs to the coumarin family, a compound known to have strong analgesic, antirheumatic, and antipyretic properties [60]. Umbelliferone was isolated for the first time from a banana flower ethanolic extract (Musa sp.) and inhibited a-glucosidase—IC_50_: 7.08 ± 0.17 μg/mL (IC_50_ of acarbose: 9.68 ± 0.48 μg/mL), inhibited α-amylase enzyme—IC_50_: 32.26 ± 1.80 μg/mL (IC_50_ of acarbose: 29.71 ± 1.51 μg/mL), and showed radical scavenging activity—EC_50__DPPH: 21.70 ± 0.37μg/mL (EC_50_ _BHA: 40.43 ± 0.81 μg/mL [61]. 

Studies showed that phenolic compounds present activity inhibitory of α-glucosidase, so more studies regarding α-amylase and α-glucosidase inhibitors have focused on the use of phenolic compounds [3,11,62].

The chlorogenic acid found in high amounts in *A. abrotanum* extracts, together with ellagic and rosmarinic acids found in high amounts in *S. officinale* extracts, showed high inhibitory activity of both enzymes, which is in agreement with the literature data [63,64,65]. The obtained experimental results recommend the two tested medicinal plants—*A. abrotanum* and *S. officinale* –as an approach to reducing post-prandial hyperglycemia.

## 4. Conclusions

The present study analyzed the chemical profile of *Artemisia abrotanum* and *Symphytum officinale* polyphenolic compounds-rich extracts obtained by membrane technologies (ultrafiltration) and their α-amylase and α-glucosidase inhibitory activity. This is the first scientific study regarding the effect of these plants in the inhibition of these enzymes.

According to the results of the present study, *Artemisia abrotanum* polyphenolic compounds-rich extracts showed high inhibitory activity of α-amylase and α-glucosidase, while *Symphytum officinale* polyphenolic compounds-rich extracts showed high α-glucosidase inhibitory activity and moderate α-amylase activity.

The obtained results showed the existence of a positive correlation between the amount of polyphenols and the antioxidant activity of the extracts, with the extracts enriched in polyphenols presenting the highest antioxidant activity through both tests used (DPPH and reducing power).

Our results regarding the correlation between chemical composition and anti-diabetic and antioxidant activities of the polyphenolic compound-rich extracts open opportunities for future research to develop nutraceuticals that possess both anti-diabetic and antioxidant activity and could be used in type 2 diabetes. 

## Figures and Tables

**Table 1:** The active biological polyphenolic compound content in the analyzed extracts.

Sample		Polyphenols (GAEμg/mL)	Flavonoids (RE μg/mL)
*A. abrotanum*	MF	723.32 ± 25.32	403.51 ± 12.59
extracts	concentrate	977.75 ± 31.67	552.85 ± 15.36
*S. officinale*	MF	667.58 ± 17.64	84.53 ± 3.2
extracts	concentrate	896.95 ± 27.21	103.21 ± 5.16

Values are expressed as mean ± SD (n = 3); GAE: gallic acid equivalent; RE: rutin equivalent.

**Table 2:** The polyphenolic compound content found in the plant extracts by HPLC.

Compound	*A. abrotanum* Polyphenolic Compounds-Rich Extract, µg/mL		*S. officinale* Polyphenolic Compounds-Rich Extract, μg/mL	
	MF	Conc.	MF	Conc.
Rutin	9.39 ± 0.23	10.57 ± 0.6	-	1.06 ± 0.06
Luteolin	1.90 ± 0.09	4.35 ± 0.08	-	1.69 ± 0.05
Quercetin	3.17 ± 0.02	5.36 ± 0.27	1.87 ± 0.04	3.42 ± 0.07
Quercetin 3-β-D-glucoside	0.29 ± 0.01	1.07 ± 0.06	-	-
Kaempferol	-	1.33 ± 0.08	1.96 ± 0.07	6.79 ± 0.11
Gallic acid	-	2.83 ± 0.06	-	-
Caffeic acid	0.58 ± 0.02	1.43 ± 0.05	7.73 ± 0.23	9.42 ± 0.24
Apigenin	1.95 ± 0.09	3.84 ±0.16	-	0.71 ± 0.02
Umbelliferone	9.59 ± 0.03	17.03 ± 0.92	3.68 ± 0.12	4.28 ± 0.13
Chlorogenic acid	85.49 ± 2.12	103.47 ± 7.21	1.45 ± 0.08	2.30 ± 0.05
Ellagic acid	-	-	37.41 ± 1.23	43.79 ± 1.89
Rosmarinic acid	-	-	120.83 ± 1.25	136.14 ± 5.16

**Table 3:** Antioxidant activity of extracts.

Samples		Reducing Power Activity EC_50_ (mg/mL)	DPPH Radical Scavenging Activity IC_50_ (µg/mL)
*A. abrotanum*	MF	521.21 ± 4.8 *	15.33 ± 1.23 *
extracts	concentrate	92.14 ± 3.2 *	9.31 ± 0.81 *
		1570.32 ± 6.5 *	17.02 ± 0.89 *
*S. officinale* extracts	MF	1091.13 ± 5.7 *	13.06 ± 0.61 *
Ascorbic acid	concentrate	1110.15 ± 4.8	11.76 ± 0.69

*The data represent the means ± SD of triplicate samples of three independent experiments.* * *p* < 0.05, reducing power compared with the polyphenols content from the extract. * *p* < 0.05, reducing power compared with the flavonoid content from the extract. * *p* < 0.05, radical scavenging activity compared with the polyphenols content from the extract. * *p* < 0.05, radical scavenging activity compared with the flavonoid content from the extract.

**Table 4:** The inhibition activity of α-amylase and α-glucosidase of analyzed extracts.

Samples		α-Amylase Inhibition IC_50_ (mg/mL)	α-Glucosidase Inhibition IC_50_ (mg/mL)
*A. abrotanum* extracts	MF	2110.12 ± 9.2 *	1450.32 ± 2.7 *
	concentrate	1881.21 ± 1.8 *	1171.16 ± 6.5 *
*S. officinale* extracts	MF	28,270.35 ± 16.7 *	413.02 ± 4.2 *
	concentrate	24,812.02 ± 12.9 *	291.56 ± 2.1 *
acarbose		1110.25 ± 8.82	372.35 ± 3.2
quercetin		351.25 ± 3.1	72.52 ± 1.5
chlorogenic acid		192.31 ± 2.3	51.21 ± 1.8
rosmarinic acid		95.63 ± 2.5	20.31 ± 0.9
kaempferol		401.32 ± 3.9	62.36 ± 2.3

* *p* < 0.05 the α-amylase inhibition activity compared with polyphenols and flavonoid contents. * *p* < 0.05 the α-glucosidase inhibition activity compared with polyphenols and flavonoid contents.

## Data Availability

The authors confirm that the data supporting the findings of this study are available within the article.
